# Seroprevalence and the Associated Risk Factors of *Neospora caninum* in Egyptian Dairy Cattle with Evaluation of Diagnostic Agreement Between Serum and Milk Samples

**DOI:** 10.1007/s11686-025-01183-z

**Published:** 2025-12-16

**Authors:** E. S. Abdel Massieh, H. M. Auda, N. M. Bakry, Reem M. Ramadan, O. H. Refaei

**Affiliations:** 1 Department of Internal Medicine and Infectious Diseases, Faculty of Veterinary Medicine, Cairo University, Giza, 12211 Egypt; 2 Department of Parasitology, Faculty of Veterinary Medicine, Cairo University, Giza, 12211 Egypt

**Keywords:** Cattle abortion, Kappa agreement, Multivariable, *Neospora caninum*, Risk factors, Seroprevalence

## Abstract

**Purpose:**

*Neospora caninum* is a common infectious cause of abortion in cattle globally. This study aimed to investigate the seroprevalence of *N. caninum*, identify associated risk factors, and assess the diagnostic agreement between serum and milk samples.

**Methods:**

A total of 254 individual serum samples were collected from dairy cattle in two Egyptian governorates. The seroprevalence of *N. caninum* antibodies was determined using a commercial ELISA kit (ID Vet – France). For assessment of diagnostic agreement between individual serum and milk samples, 92 milk samples from the same animals representing different days in milk (DIM) were tested using the same ELISA kit.

**Results:**

The overall seroprevalence of *N. caninum* antibodies was 31.9%. The seroprevalence rates for Faiyum and Alexandria governorates were 28% and 40.5%, respectively. For seropositive animals to *N. caninum*, the odds of suffering from abortion are 5.5 times greater than the odds for seronegative animals (*P* < 0.001), reaching 12.4 times in multivariable analysis. While the animal’s location was found to have a significant relationship with the seroprevalence of *N. caninum* (*P* = 0.048) in univariable analysis, multivariable logistic regression showed no significant effect (*P* = 0.33*)*. The age, parity, animal productive category, gynecological disorders, and the average milk production had no significant relation with the seroprevalence of *N. caninum* (*P* > 0.05). The Kappa coefficients between individual serum and milk samples were 0.59, 0.52, and 0.64 for all samples (n = 92), ≤ 220 DIM (n = 42), and ˃ 220 DIM (n = 50), respectively.

**Conclusion:**

*N. caninum* is prevalent in Egypt. Abortion history can be used as a key predictor factor for *Neospora* in the examined herds. The late lactation period is the preferred time for testing milk samples; however, we do not recommend replacing individual serum samples with milk samples.

## Introduction

Neosporosis, caused by the protozoan parasite *Neospora caninum*, is primarily a disease of cattle and dogs [1]. It is one of the most significant protozoan infectious causes of abortion in cattle internationally [2–4]. Abortion is considered one of the most significant causes of economic losses in dairy farms worldwide [5, 6]. Economic losses are related to losses of newborn calves and future replacement heifers, feeding costs, milk production losses, and costs of additional insemination [7].

Dogs serve as the definitive hosts for *N. caninum*; they shed oocysts in their feces, while cattle, as an intermediate host, acquire the infection either horizontally through the consumption of feed and water contaminated with sporulated oocysts or vertically through transplacental transmission, which is regarded as the primary route for cattle infection [8–10]. *Neospora* infection in cattle is mainly associated with early embryonic death, abortion, perinatal mortality, or parturition of a deformed calf [11]. The pattern of abortion associated with neosporosis in the herd is usually related to the route of transmission; in the case of the epidemic pattern, the horizontal route of transmission is the prevailing route, while in the endemic pattern, the vertical transmission is the dominant route [12].

There are several risk factors associated with *N. caninum* infection in cattle, including the virulence of *N. caninum*, routes of transmission (vertical or horizontal), type of infection either primary infection, recrudescence, or reinfection, and the stage of pregnancy during which the dam is infected [13]**.** Other risk factors of neosporosis may be attributed to the origin of the animals, breeds, age, and exposure to dogs or contaminated feed [14]. Furthermore, it is assumed that climatic factors such as rainfall and minimal average temperatures promote the survival and sporulation of oocysts [2, 15].

Several diagnostic techniques have been developed to facilitate the diagnosis of this parasitic infection [10]. The diagnostic criteria for *N. caninum*-induced abortion are the identification of specific histopathological lesions, detection of parasites in fetal tissues by Polymerase chain reaction (PCR) or Immunohistochemistry (IHC), and detection of specific antibodies in serum using serological tests [11]. Conventional serological techniques, mainly indirect fluorescent antibody test (IFAT) and enzyme-linked immunosorbent assay (ELISA) are most frequently employed in adult animals to identify antibodies in serum samples [3]. Currently, ELISA technique is the most widely used serological test due to the ease of application and the ability to analyze multiple samples in the same assay, allowing for rapid and inexpensive screening of neosporosis infection in dairy farms [16].

The detection of specific antibodies in serum samples is employed in the routine diagnosis of *N. caninum* infection in cattle, but milk samples can be used for lactating cows [11]. Milk ELISA can represent a valuable tool for screening and monitoring *N. caninum* prevalence in dairy farms [3, 17]. The simplicity of milk sampling as well as the reduction of stress-related production loss are considered the main benefits for detecting antibodies from milk samples in dairy animals [3, 11]. However, the main drawback of utilizing milk samples in serological diagnosis is that males, non-lactating, and dry animals are not included in the sampling [18].

As cattle are the main source of red meat and milk for human consumption in Egypt with an animal population exceeding 5 million; many studies have investigated different infectious agents, causing abortion in cattle including neosporosis [19, 20]. Identification of *N. caninum* epidemiology, potential risk factors, and a reliable diagnostic approach are critical for implementing control and prevention strategies due to the lack of an appropriate therapy or vaccination for neosporosis [10, 11, 21]. Therefore, the primary objective of this study is to determine the seroprevalence of *N. caninum* in dairy herds in two different Egyptian governorates, determine its associated risk factors, and identify the appropriate sample for the serological diagnosis of neosporosis based on the evaluation of the agreement between individual serum and milk samples.

## Materials and Methods

### Study Area

Our cross-sectional study was conducted in two geographically and climatically distinct Egyptian governorates: Alexandria in Lower Egypt and Faiyum in Upper Egypt. Alexandria is located on the Mediterranean coast and is characterized by moderate temperature and higher humidity [22]. In contrast, Faiyum is inland and is characterized by a dry, arid climate with lower humidity [23]. The targeted cattle population in both governorates was the medium and large organized commercial dairy farms that had accurate data records. Breeding of cows is primarily via artificial insemination. There is no implemented routine screening test for different infectious diseases in these areas.

### Ethical Approval

The Institutional Animal Care and Use Committee of the Faculty of Veterinary Medicine Cairo University (Vet. CU. IACUC) approved all methods, including the handling of animals for blood and milk samples collection in this study, with approval number Vet CU 01122022607.

### Animals and Sampling

**Fig. 1:**
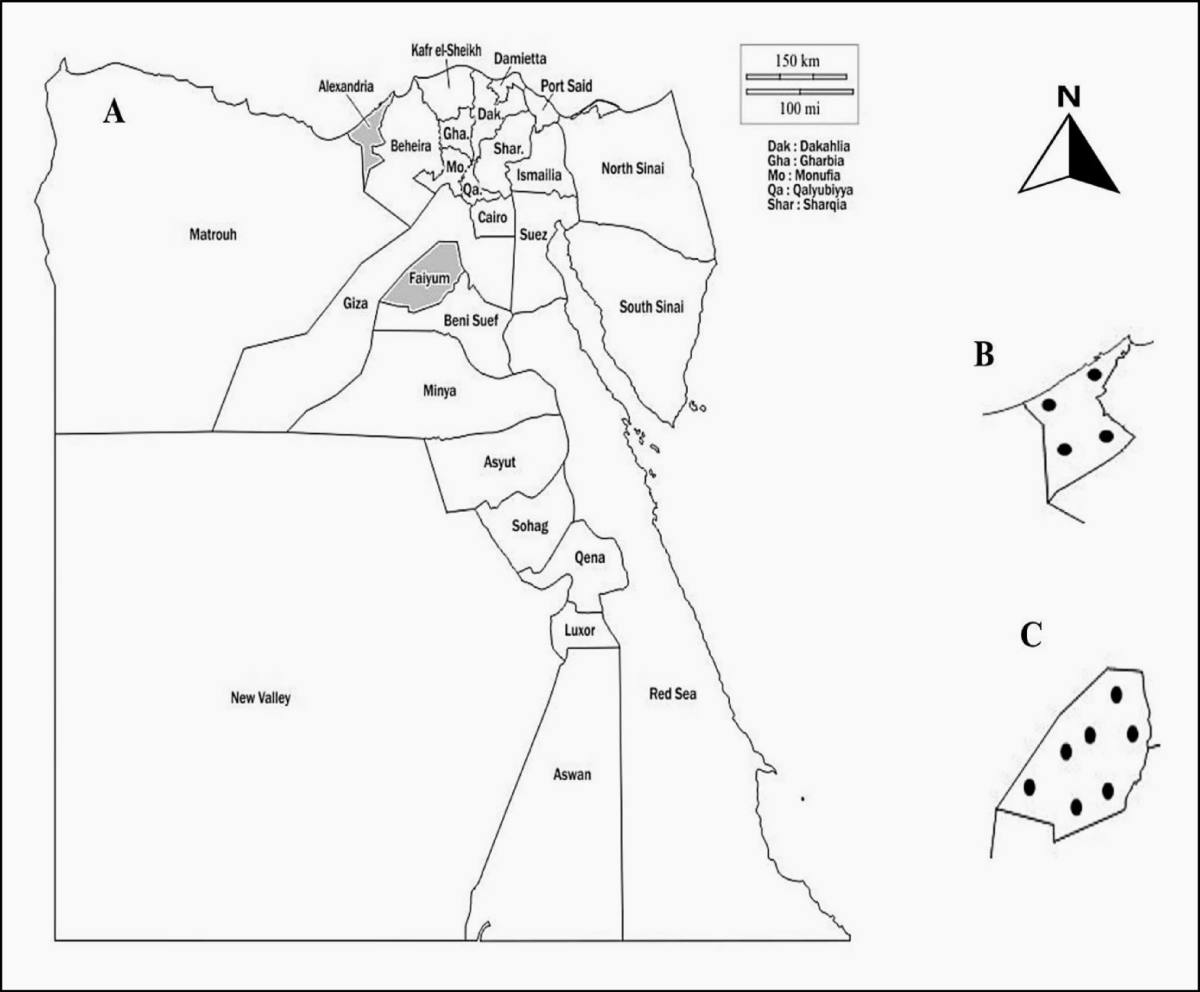
**A** Egypt map, the shadow areas represent the two sampled governorates: Alexandria and Faiyum; **B** represents the Alexandria governorate map and **C** represents Faiyum governorate map. The symbol (●) shows the approximate locations of the examined herds in the two governorates.

A total of 254 dairy cattle were selected and sampled from two different Egyptian governorates (Fig. 1), Alexandria governorate in Lower Egypt (n = 79) and Faiyum governorate in Upper Egypt (n = 175). Eleven dairy herds from various localities in Alexandria (n = 4) and Faiyum (n = 7) governorates were selected based on the presence of abortion history, and at least 10% of cattle aged more than one year were randomly selected and sampled from each herd.

Blood samples were collected from selected animals between January and December, 2023, serum was separated and stored at − 20 °C until serological testing.

For evaluation of the diagnostic agreement between serum and milk samples, 221 milk samples were collected from lactating cows selected from the serum-sampled animals, with both samples obtained at the same time . A total of 33 animals were excluded from milk sampling due to either suffered from clinical mastitis, were dry cows, or were heifers during the sampling time. Milk samples were centrifuged at 252 × g for 10 min; the lactoserum was collected from each sample beneath the creamy layer and stored at − 20 °C until serological testing.

### Serological Testing

Detection of *N. caninum* antibodies in serum and milk samples was performed using the commercial ID Screen® *Neospora caninum* Multispecies Indirect ELISA kit (ID Vet – France) according to the manufacturer’s instructions. The sample-to-positive percentage (S/P %) was calculated using the following equation:

$$S/P\%=\left(\frac{{OD}_{sample}-{OD}_{NC}}{{OD}_{PC} -{OD}_{NC} }\right) \times 100$$

where NC is the negative control and PC is the positive control. For serum samples, S/P % ≤ 40% is considered negative, 40% < S/P % < 50% doubtful, and S/P % ≥ 50% positive. For milk samples, S/P % ≤ 25% is considered negative, 25% < S/P % < 30% doubtful, and S/P % ≥ 30% positive.

### Data Collection and Animals Grouping

The collected data from the sampled animals were classified according to their location (Alexandria and Faiyum), age (< 4 years, 4–6 years, and > 6 years), number of parities (first, second, and three or more parities), productive category (heifers and cows), daily milk production (low: ≤ 15 kg/day, medium: > 15–25 kg/day, and high: > 25 kg/day), history of abortion (yes or no), number of abortions (1 and ≥ 2), the season of abortion (winter and summer), stage of abortion either early (from 45 days to 6 months of gestation) or late (from 7 months till the entire term of gestation), history of reproductive problems including embryonic death, low conception rate, and infertility (yes or no), and days in milk (DIM) for lactating cows (≤ 220 DIM represent early-mid lactation period and ˃ 220 DIM represent late lactation period). All sampled cattle were females and Holstein breed.

### Univariable and Multivariable Analysis

Data were presented as percentages and a 95% confidence interval (CI). The Chi-square (χ^2^) test and Fisher’s Exact test (FET) were employed to examine the relationship between different variables and *N. caninum* seroprevalence. Statistical significance was defined as a *P*-value < 0.05. The odds ratio (OR) for different variables was also estimated. The baseline group for all variables is illustrated in (Table 1).

**Table 1:** Seroprevalence rate of *Neospora caninum* in cattle regarding different variables

Variable	Categories	Total of examined cattle	N° of seropostive	Seropositive % (95%CI)	OR (95%CI)	*X*^*2*^ value (*P* value)^a^
Abortion occurrence	Yes	89	50	56.2 (45.8, 66.0)	5.54 (3.13, 9.82)	37.216 *P* < 0.001
	No	165	31	18.8 (13.6, 25.6)	1.0 (baseline group)	
Abortion number	1	71	37	52.1 (40.7, 63.3)	0.42 (0.14, 1.3)	2.359 (*P* = 0.125)
	≥ 2	18	13	72.2 (48.8, 87.8)	1.0 (baseline group)	
Last abortion season	Winter	58	33	56.9 (44.1, 68.8)	1.09 (0.45, 2.61)	0.035 (*P* = 0.852)
	Summer	31	17	54.84 (37.77, 70.85)	1.0 (baseline group)	
Abortion stage	Early	43	26	60.5 (45.6, 73.7)	0.85 (0.32, 2.28)	0.105 (*P* = 0.746)
	Late	28	18	64.3 (45.8, 79.4)	1.0 (baseline group)	
Reproductive problems	No	227	71	31.3 (25.6, 37.6)	0.77 (0.34, 1.77)	0.369 (*P* = 0.544)
	Yes	27	10	37.0 (21.5, 55.8)	1.0 (baseline group)	
Average milk production/ day	Low	24	5	20.8 (8.8, 40.9)	0.51 (0.18, 1.43)	3.985 (*P* = 0.136)
	Medium	155	53	34.2 (27.2, 41.97)	1.0 (baseline group)	
	High	51	11	21.6 (12.3, 34.8)	0.53 (0.25, 1.11)	
Location	Faiyum	175	49	28 (21.9, 35.1)	1.0 (baseline group)	3.919 (*P* = 0.048)
	Alexandria	79	32	40.5 (30.4, 51.5)	1.75 (1.00, 3.06)	
Age (years)	< 4	98	30	30.6 (22.3, 40.3)	0.77 (0.39, 1.5)	0.828 (*P* = 0.661)
	4–6	93	28	30.1 (21.7, 40.1)	0.75 (0.38, 1.47)	
	> 6	63	23	36.5 (25.7, 48.9)	1.0 (baseline group)	
Parities number	1	44	15	34.1 (21.8, 48.9)	1.79 (0.77, 4.18)	3.108 (*P* = 0.211)
	2	67	15	22.4 (13.96, 33.8)	1.0 (baseline group)	
	≥ 3	129	44	34.1 (26.5, 42.7)	1.79 (0.91, 3.54)	
Productive category	Cow	240	74	30.8 (25.3, 36.9)	0.45 (0.13, 1.55)^*^	*P* = 0.148^*^
	Heifer	14	7	50.0 (26.8, 73.2)	1.0 (baseline group)	

^**a**^The result is significant at *P* < 0.05

^*^Fisher exact test is used rather than chi-square as at least one expected value is < 5

To further clarify the factors associated with *Neospora* seropositivity, a multiple logistic regression model was developed by incorporating variables that showed significant or near-significant associations in the univariable analyses.

Statistical analyses and calculations were conducted using JASP (Version 0.19.0) [Computer software, 2024. Available from: https://jasp-stats.org/].

Receiver operating characteristic (ROC) curve for the multiple logistic regression model predicting *Neospora* seropositivity based on ELISA serum results is plotted by calculating and graphing pairs of True Positive Rate (TPR) (y-axis) and False Positive Rate (FPR) (x-axis). The (ROC) curve is performed and plotted by JASP (Version 0.19.0).

### Agreement Between Serum and Individual Milk Samples

The agreement between the ELISA results of serum and individual milk samples of the same animals (n = 92) were selected from the collected 221 milk samples, the 92 selected milk samples representing different days in milk (DIM) and positive and negative serum samples results. The degree of agreement was calculated by the Kappa coefficient and assessed using the Landis and Koch scale [24] to determine the possibility of replacing serum samples with individual milk samples. The interpretation of the Kappa result is as follows: values ≤ 0 indicating poor agreement, 0.01–0.20 as slight, 0.21–0.40 as fair, 0.41–0.60 as moderate, 0.61–0.80 as substantial, and 0.81–1.00 as almost perfect agreement.

The Kappa coefficient agreement calculation was performed using SPSS software, version 24 (IBM®, SPSS® Inc., Chicago, USA).

## Results

### Seroprevalence of *N. caninum*

The overall seroprevalence rate was 31.9% (81/254, CI 95%: 26.5–37.9%). All the examined 11 herds were positive for *N. caninum* antibodies. In Alexandria governorate, the seroprevalence was 40.5% (32/79, CI 95%: 30.4%–51.5%). This was higher than that of Faiyum governorate, which was 28% (49/175, CI 95%: 21.9%–35.1%).

### Univariable Analysis of Different Variables Associated with *N. caninum* Seroprevalence

The occurrence of abortion was significantly associated with *Neospora* seropositivity (*P* < 0.001) (Table ); the odds of suffering from abortion in seropositive animals were 5.5 times greater than the odds in seronegative animals, with OR (95% CI) equals 5.5 (3.1, 9.8). However, the Chi-square test of independence and the OR indicated no significant association between *Neospora* serostatus and the number, season, and stage of abortion (*P* > 0.05) (Table 1). Furthermore, no significant associations were detected for the presence of reproductive problems and average daily milk production (all *P* > 0.05) and *N. caninum* seropositivity. Regarding other different variables associated with *Neospora* animal serostatus, location had a potential impact on *N. caninum* seropositivity (*P* = 0.048). In contrast, there was no significant relationship between different age groups (*P* = 0.661), parity groups (*P* = 0.211), and animal productive categories (*P* = 0.148) with *Neospora* seroprevalence (Table 1).

### Multivariable Analysis: Independent Predictors and Model Performance

To further clarify the factors associated with *Neospora* seropositivity, a multiple logistic regression model (Table 2) was developed by incorporating variables with (*P* values ≤ 0.3) in the univariable analyses (Table 1) except animal reproductive category due to class imbalance (240 cows vs. 14 heifers) and missing data patterns from other covariates.

**Table 2:** Associations between different factors and *Neospora caninum* seropositivity (multiple logistic regression)

Variable	OR	*P*-Value	95% CI lower	95% CI upper
Intercept	0.167	< 0.001	0.076	0.368
Location = “Alexandria”	0.65	0.33	0.27	1.56
Parities number = 1	1.92	0.18	0.73	5.04
Parities number = " ≥ 3"	1.89	0.12	0.84	4.26
Average milk production/ day = "High"	0.70	0.39	0.31	1.58
Average milk production/ day = "Low"	0.39	0.11	0.12	1.25
Abortion occurrence = "Yes"	12.41	< 0.001^*^	2.99	51.48
Abortion numbers = 1	0.54	0.38	0.14	2.14
Abortion numbers = “ ≥ 2”^**†**^	–	–	–	–

†Could not be estimated due to quasi-complete separation (insufficient observations in ≥ 2 abortions group). *Indicates statistical significance (*P* < 0.05)

Following adjustment for potential confounders, a history of abortion emerged as a strong independent predictor of *Neospora* seropositivity (OR = 12.4, 95% CI 3–51.5, *P* = 0.0005), thereby highlighting a robust association even when accounting for other variables. In contrast, herd location (Alexandria vs. Faiyum; OR = 0.65, 95% CI 0.27–1.56, *P* = 0.33) did not maintain statistical significance with *Neospora* seropositivity.

**Fig. 2:**
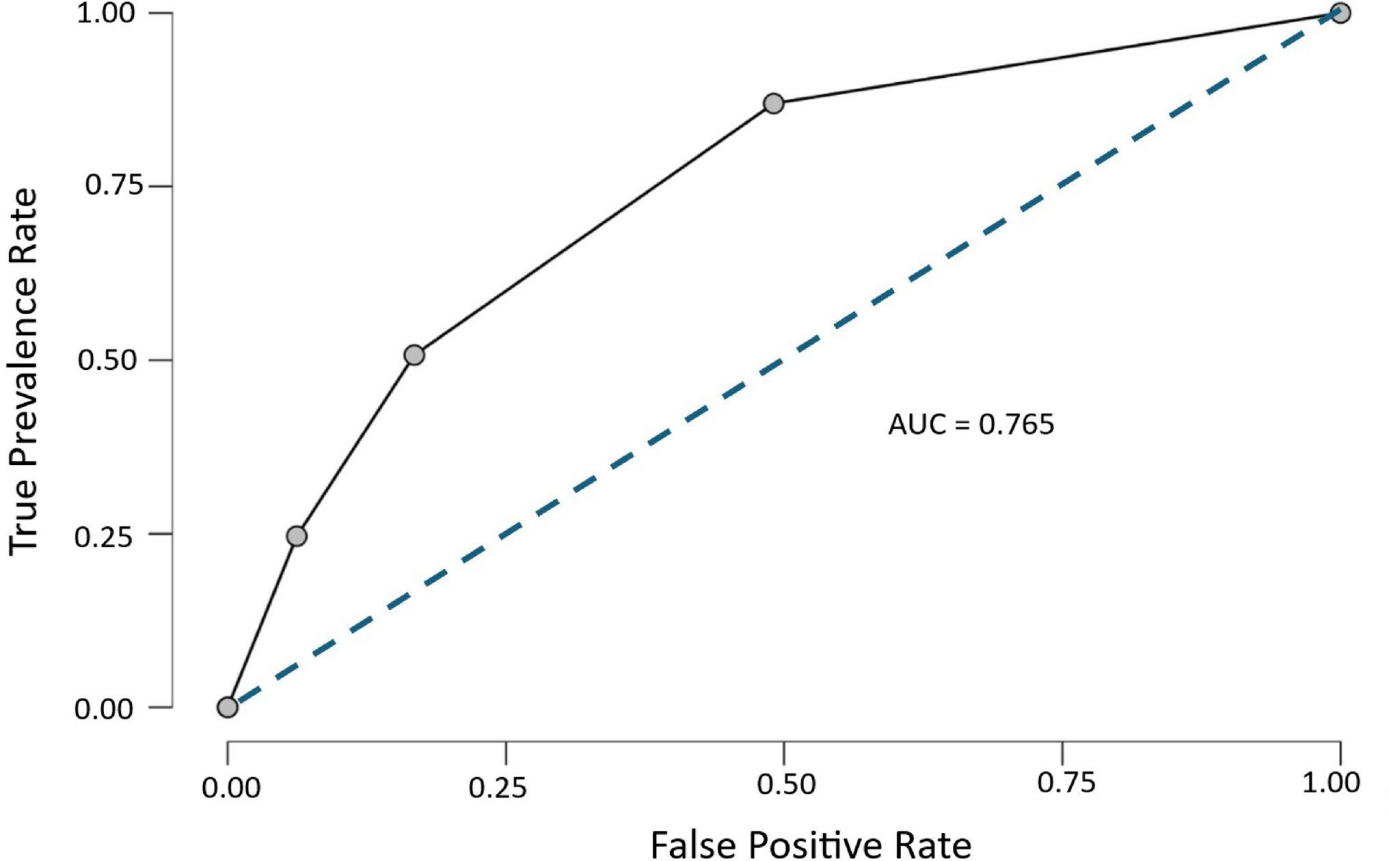
Receiver operating characteristic (ROC) curve for the multiple logistic regression model predicting *Neospora* seropositivity based on ELISA serum results. The model achieved an area under the curve (AUC) of 0.765, indicating good discriminatory ability

Furthermore, the model demonstrated good discriminatory power for ELISA serum status, as evidenced by an area under the ROC curve (AUC) of 0.765 (Fig. 2). Taken together, these results underscore the pivotal role of abortion history as a key predictor factor for *Neospora* seropositivity and emphasize the utility of multivariable analysis in identifying independent predictors within epidemiological datasets.

### Agreement Between Individual Serum and Milk Samples for Detection of *N. caninum* Antibodies

The Kappa agreement between the results of serum samples (n = 92) and corresponding milk samples when both tested by the same ID ELISA kit was 0.59; according to the Landis and Koch scale, the degree of agreement was considered moderate. The kappa value between results of serum and corresponding milk samples concerning days in milk (DIM) was 0.52 and 0.64 for ≤ 220 DIM (n = 42) and ˃ 220 DIM (n = 50), respectively; with moderate degree of agreement (Table 3).

**Table 3:** Kappa value, degree of agreement between serum and milk samples, and number of tested milk samples for detection of *Neospora caninum* antibodies using ELISA kit

Agreement measure	Number of tested samples	Kappa value	*P* value	Agreement degree
Over all kappa agreement	92	0.59	< 0.0001	Moderate agreement
≤ 220 DIM	42	0.52		
˃ 220 DIM	50	0.64		

## Discussion

Besides the challenges faced by many African countries due to neosporosis [5, 6, 25], *Neospora caninum* has extensively spread worldwide in Asia [26, 27], Europe [9, 16, 17, 28], and America [29–32]; with recently recorded abortion outbreaks due to neosporosis in many countries [30, 32].

The overall seroprevalence reported in this study (31.9%) closely aligns with previous findings in cattle in Egypt. For instance, Fereig et al*.* [20] and Selim et al. [10] reported overall *Neospora* seroprevalence rates of 33.3% and 28.9%, respectively. Other studies in Egypt recorded lower seroprevalence rates than those observed in our study, including 24.6% [19], 15.5% [33], and 18.9% [34]. The study protocol, including study design, animal grouping and sampling, diagnostic techniques, as well as hygiene and feeding systems, may affect the seroprevalence and the significance of the associated risk factors across different studies [9, 10, 19].

The relationship between the risk of abortion and the animal serological status of *Neospora* revealed a substantial correlation between an increased likelihood of abortion and *Neospora* infection. It has been demonstrated that cows seropositive for *N. caninum* are more likely to suffer miscarriages than seronegative ones [8, 35]. Prior studies reported that the risk of abortion was 2–26 times higher in seropositive cows than in seronegative ones [11]. For instance, Martinez et al. [36] determined that seropositive cows had a 6.63-fold higher risk of abortion than seronegative cows, emphasizing the significance of *N. caninum* as a cause of abortion in dairy cattle. A further study revealed that the percentage of seropositive cows with an abortion history was significantly greater than the percentage of seronegative cows [10]. On the contrary, Abdeltif et al. [25] revealed no statistically significant correlation between *N. caninum* seropositivity and abortion in Algerian pregnant cows, either at the farm or individual level.

Consistent with the findings of Cardoso et al. [37], cattle suffering from repeated abortions did not exhibit a notably increased seropositivity for *N. caninum* infection. This may be attributed to the fact that most animals gained immunity after the first abortion, leading to a decrease in the rate of subsequent abortion [8, 38]. Furthermore, no association was detected between *Neospora* seroprevalence and the season of abortion. A similar finding was reported in Spain [39]. However, previous studies have indicated significant variation between *Neospora* infection and abortion season, which is influenced by climatic differences between countries and even within different regions of the same country [15]. For instance, Nasir et al. [40] found that abortions associated with *N. caninum* are more prevalent during the summer, while Wei et al. [27] recorded that *Neospora*-related abortion is more common in spring and autumn.

Although abortion induced by *N. caninum* most commonly occurs at 5–6 months of gestation [41], it can also occur early during the second trimester till the end of the gestation period [16]**.** This may explain our finding of no significant association between *Neospora* seropositivity and different stages of abortion, whether early or late.

The reproductive disorders in the investigated animals were not significantly associated with *Neospora* seroprevalence. A comparable study revealed no distinct relation between *Neospora* and reduced fertility [17]. Moreover, López-Gatius et al. [39] reported that *Neospora* infection had no adverse effect during the early fetal period. These results verify the theory that the majority of abortions correlated with neosporosis occur in the second and third trimesters when the cellular immunity is impaired [42, 43]. Contrary to these findings, a prior study demonstrated considerable seropositivity for *N. caninum* infection in cattle suffered from repeat breading and early embryonic death [10]. Moreover, Lefkaditis et al. [44] demonstrated a prolonged calving to conception interval and an increased number of inseminations in seropositive cows.

In agreement with Hobson et al. [45], this study found no effect of *Neospora* infection on milk yield. A comparable study demonstrated an increase in milk production in seropositive cows [46]. Furthermore, Villa et al. [12] reported that the seropositive cows in one farm produced low milk per day and had a lower mature equivalent milk production, while seropositive cows in another farm exhibited higher levels of both parameters than seronegative animals. It is challenging to determine whether neosporosis significantly impacts productive parameters since milk production is dependent on a variety of factors, including farm influence, which is the culmination of genetics, management, and environmental factors [47].

Regarding the well-recognized risk factors associated with *Neospora* infection, including animal breed and purpose of production, housing and feeding systems, and general biosecurity measures [6, 9, 19]. Animals’ location was supposed to affect the seroprevalence of *Neospora*. Alexandria governorate is characterized by a wet climate and high soil moisture, which may favor *N. caninum* viability in contrast to Faiyum governorate [2, 22, 23]. The univariable analysis reveals a significant association between animal location and *Neospora* seropositivity, however the multivariable logistic regression showed no significant correlation, suggesting that the location effect may be confounded or less influential relative to abortion history. Climate conditions can affect the survival of *N. caninum* oocysts, but do not directly influence seroprevalence in cattle. Moreover, other factors correlated to animal management and sanitary practices may play a more significant role [48]. In contrast to our finding, Semango et al. [5] and Abdeltif et al. [25] recorded a significant association between different locations and *N. caninum* seroprevalence rates.

The current study demonstrated no statistically significant association between age and *Neospora* seroprevalence, which is consistent with prior findings [49–52]. Age may have an inverse or non-significant influence on seropositivity in areas where vertical transmission is more predominant, as immunity wanes over time due to lower intermittent exposure [14, 53]. However, several studies have found that older dairy cattle are more likely to be seropositive which may be attributed to horizontal transmission and cumulative exposure [10, 14, 16, 25, 54]. Furthermore, there was no significant difference in seroprevalence concerning parity; the same results were recorded by Selim et al. [10] and González-Warleta et al. [17] while disagreeing with Miroud et al. [55], who reported that *Neospora* seroprevalence increases with parity. Regarding the animal productive category, no significant correlation was detected in this study between cows and heifers which is consistent with a prior study [12].

Assessment of diagnostic test accuracy for neosporosis in cattle is difficult as there is no gold standard technique [11]. The main challenge facing the serological diagnosis of *N. caninum* using ELISA is the potential for false-positive and false-negative results [56]. Moreover the type of ELISA kit used and the type of the utilized coated antigens may influence the ELISA results [57].

In this study, the agreement between the results of individual serum and milk samples using the ELISA test was detected using the kappa coefficient to judge the potential of replacing individual serum samples by milk samples. The kappa agreement between the two samples was 0.59, with moderate agreement according to the Landis and Koch scale [24]. This finding is nearly similar to previous studies, which detected Kappa values at 0.52 and 0.568 in cows [29] and buffaloes [58], respectively. Our findings also agreed with Bezerra et al. [59], who found the kappa value between serum and colostrum of ewes was 0.558. In contrast, Fereig et al. [60] and Enachescu et al. [61] detected higher kappa values of 0.7 and 0.72, respectively, indicating substantial agreement strength. Moreover, Cirone et al. [3] and González-Warleta et al. [17] detected Kappa values at 0.86 and 0.908, respectively, with agreement strength as almost perfect. A significant diversity in the kappa agreement value across different studies can be related to the fact that the antibody titer of *Neospora caninum* is detected later in milk and is approximately 30 times lower in concentration compared to serum [11]. Other causes of the kappa value difference may be the different sensitivity of commercial diagnostic kits to milk samples and technical errors when using milk samples.

For selecting the preferred time for milk sampling in serological testing of *N. caninum*, the kappa agreement between serum and milk samples was determined concerning the lactation period, as ≤ 220 DIM and ˃ 220 DIM. The values of kappa agreement were 0.524 and 0.639 for ≤ 220 DIM and ˃ 220 DIM, respectively. The higher value of kappa agreement at the late lactation period is compatible with the increase in the *Neospora caninum* antibody titer in the second half of the gestation period in pregnant lactating cows [62].

In conclusion, *N. caninum* is prevalent in Egypt, with a significant association observed between the seroprevalence of *Neospora* and the abortion rate. Abortion history can be used as a key predictor factor for *Neospora* in the examined herds. Multivariable analysis is pivotal in the detection of predictor factors for *Neospora* infection. Based on our findings, the late lactation period is the preferred time for testing milk samples. However, we do not recommend replacing individual serum samples with milk samples because of the moderate agreement result between serum and milk samples. Therefore, a more reliable and specific serological test for the detection of *N. caninum* antibodies in milk is required.

Additional studies are required to investigate the seroprevalence of *N. caninum* in various locations throughout Egypt. It is also crucial to thoroughly assess how management and sanitary practices impact the prevalence of *N. caninum*, intending to implement targeted prevention and control measures.

## Data Availability

The data that support the findings of this study are available in the published article.
